# A cellulose nanofibril-reinforced hydrogel with robust mechanical, self-healing, pH-responsive and antibacterial characteristics for wound dressing applications

**DOI:** 10.1186/s12951-022-01523-5

**Published:** 2022-07-06

**Authors:** Guihua Yang, Zhikun Zhang, Kefeng Liu, Xingxiang Ji, Pedram Fatehi, Jiachuan Chen

**Affiliations:** 1 State Key Laboratory of Biobased Material and Green Papermaking, Qilu University of Technology (Shandong Academy of Sciences), Jinan, 250353 Shandong China; 2 Biorefining Research Institute and Chemical Engineering Department, Lakehead University, Thunder Bay, ON Canada

**Keywords:** Cellulose nanofibril, Hydrogel, Resveratrol, pH-responsive, Wound healing

## Abstract

**Background:**

Bacterial infection in wounds has become a major threat to human life and health. With the growth use of synthetic antibiotics and the elevated evolution of drug resistant bacteria in human body cells requires the development of novel wound curing strategies. Herein, a novel pH-responsive hydrogel (RPC/PB) was fabricated using poly(vinyl alcohol)-borax (PB) and natural antibiotic resveratrol grafted cellulose nanofibrils (RPC) for bacterial-infected wound management.

**Results:**

In this hydrogel matrix, RPC conjugate was interpenetrated in the PB network to form a semi-interpenetrating network that exhibited robust mechanical properties (fracture strength of 149.6 kPa), high self-healing efficiency (> 90%), and excellent adhesion performance (tissue shear stress of 54.2 kPa). Interestingly, the induced RPC/PB hydrogel showed pH-responsive drug release behavior, the cumulative release amount of resveratrol in pH 5.4 was 2.33 times than that of pH 7.4, which was adapted well to the acidic wound microenvironment. Additionally, this RPC/PB hydrogel exhibited excellent biocompatibility and antioxidant effect. Moreover, in vitro and in vivo results revealed that such RPC/PB hydrogel had excellent antibacterial, skin tissue regeneration and wound closure capabilities.

**Conclusion:**

Therefore, the generated RPC/PB hydrogel could be an excellent wound dressing for bacteria-infected wound healing.

**Supplementary Information:**

The online version contains supplementary material available at 10.1186/s12951-022-01523-5.

## Introduction

As the immune system's first line of defense, the skin serves an important function in defending the human body from external invaders. However, skin is often at risk of bacterial infection after injury, especially staphylococcus aureus (*S. aureus*), which accounts for nearly 60% of all bacterial infections [1–3]. Generally, the wound healing process involves four steps: hemostasis, inflammation, proliferation, and rebuild [4, 5]. Bacterial infection will disrupt these phases, and the delayed wound healing will result in chronic infection at the wound site, causing the infection to worsen [6]. Traditional dressings, such as various gauze and bandage, are commonly used materials in the wound treatment because of their low cost, wide source of raw materials, soft texture, and strong absorption capacity readily preventing the accumulation of wound seepage [7–9]. Unfortunately, these traditional dressings have many drawbacks, such as limited ability to promote wound healing and poor moisturizing effect, resulting in adhesion and scab of dressing in the wound site. Also, when these dressings penetrate into the wound tissue, they can cause exogenous infection. Moreover, such traditional dressings have a marginal effect on wounds that are infected with bacteria [10]. Thus, it is of great significance to develop a bioactive dressing that can rapidly reduce wound site infection and promote wound healing.

Hydrogels encapsulated with antibacterial agents are promising formulations for bacterial-associated wounds due to their adjustable physicochemical properties and softness similarities to extracellular matrixes [11–14]. Additionally, hydrogel dressings can form physical barriers at wound area to help in hemostasis, keep the wound moist, and enable oxygen to pass through [15, 16]. Synthetic antibiotics are the most commonly used drugs to treat wound infections, but overuse may lead to drug resistance and long-term adverse effect [17, 18]. Interestingly, many studies have found that natural antibiotics (e.g., flavonoids, alkaloids, phenolic compounds, proteins, and organic acids) exhibited excellent antibacterial, antiviral, and anti-inflammatory ability, but less burden on the human body [19–24]. Also, natural antibiotics contains a variety of vitamins, minerals and biological activity, which may facilitate human body's immune system and resist bacteria attack [25, 26]. Resveratrol (RSV), as a non-flavonoids polyphenol compound, has good anticancer, anti-oxidation, antibacterial, anti-inflammatory properties and low drug resistance. At present, the study of RSV mainly focused on the field of the anti-tumor, and there are few reports about wounds treatment [27–30]. Thus, hydrogels loaded with RSV to treat wound infections would be a promising strategy.

A major challenge in producing hydrogels for antibiotic delivery is the generation of hydrogels with the controllable release of antibiotics since excessive release of antibiotics can lead to severe systemic toxicity and irreversible damage to the patient's health while low release can cause drug resistance [31–33]. As is well known, the wound microenvironment will become acidic with bacteria proliferation [34, 35]. Therefore, it may be necessary to develop hydrogel with self-adjusting antibiotic release according to pH of the environment. Ideally, this pH-responsive hydrogel will degrade when bacteria proliferate, and then release the antibiotics to defeat bacteria; while the degradation process will be terminated when the pH of the wound area returns to normal. Poly(vinyl alcohol)/borax (PVA/borax) hydrogel has been widely studied for biomedical applications [36–38]. Taking use of the dynamic borate ester in hydrogel network, PVA/borax hydrogel is able to break and rebuild in response to pH changes, and thus achieving the goal of controlled drug release [39–41]. However, a serious drawback of PVA/borax hydrogel as wound dressing is its poor mechanical strength, which cannot provide effective protection for the wound [42–44]. Adding polymers to hydrogel system to construct interpenetrating networks (IPN) or semi-interpenetrating network (semi-IPN) is a viable way for improving the mechanical strength of hydrogels [45–49]. Cellulose nanofibrils (CNFs), as a strong biobased nanomaterial with unique mechanical properties, ideal biocompatibility and abundant active groups, has received tremendous attentions and is widely used for various field [50–57]. To our knowledge, the integration of antibiotic grafted CNF in the PVA/borax system for the hydrogel production and the impact of CNF on the performance of the hydrogel have not been studied.

In this work, a novel pH-responsive CNF-reinforced PVA/borax hydrogel was fabricated, which was crosslinked by dynamic borate bonds and hydrogen bonds. Furthermore, CNFs were grafted with the natural antibiotic RSV to equip the hydrogel with potent antibacterial and antioxidant properties. The physicochemical properties of the fabricated hydrogel, including the structure, mechanical strength, rheological properties, self-healing ability, swelling ratio and water vapor permeability were characterized. Moreover, the in vitro pH-responsive RSV release behavior, biocompatibility and antioxidant performance of the induced hydrogel were investigated. Finally, the in vitro antibacterial ability, in vivo wound healing efficiency and tissue regeneration ability of the hydrogel were evaluated systematically.

## Materials and methods

### Materials

Tempo-oxidized cellulose nanofibrils (TOCNFs) with the carboxyl content of 1.4 mmol/g were prepared in a laboratory setting, and the preparation process is expressed in the Additional file 1. NH_2_-PEG-COOH (M_W_ = 2000), resveratrol (RSV), poly(vinyl alcohol) (PVA), borax, phosphate buffer solution (PBS), Triton X-100, 1,1-diphenyl-2-picrylhydrazyl free radicals (DPPH·), ethanol and Tegaderm (3 M) were provided by Sinopharm Chemical Reagent Co., Ltd. (Beijing, China). N-hydroxysuccinimide (NHS), 4-Dimethylaminopyridine (DMAP), 1-(3-Dimethylaminopropyl)-3-ethylcarbodiimide hydrochloride (EDC), paraformaldehyde, paraffin, and 3-(4,5-dimethyl-2-thiazolyl)-2,5-diphenyl-2-H-tetrazolium bromide (MTT) were obtained from Sigma-Aldrich. Regenerated cellulose dialysis membranes, Luria–Bertani (LB) broth containing tryptone (10 mg/mL), NaCl (10 mg/mL) and yeast extract (5 mg/mL) were obtained from Aladdin Co., Ltd (Shanghai, China).

Mouse fibroblast cells (L929) and staphylococcus aureus (*S. aureus*, 25923) were provided by ATCC. Kunming (KM) mice were obtained from experimental animal center of Shandong University of Traditional Chinese Medicine. All animal experiments were carried out in accordance with Shandong University of Traditional Chinese Medicine's animal care and experiment rules.

### Synthesis of RSV-PEG-CNF conjugate

The resveratrol-polyethylene glycol-cellulose nanofibrils (RSV-PEG-CNF, RPC) conjugate was synthesized via a two-step process. In the first step, RSV (0.034 g, 0.15 mmol) was dissolved in 70 mL trichloromethane. After dissolving, NH_2_-PEG-COOH (0.20 g, 0.10 mmol), EDC (0.023 g, 0.12 mmol) and DMAP (0.014 g, 0.12 mmol) were added to the system, and the mixture was stirred for 24 h at 25 °C. After the reaction, the solvent was removed from the system by rotary evaporator (N-1300 V, EYELA, Japan). Then, the unreacted catalyst (i.e., EDC and DMAP) and excess amount of RSV were removed from the reaction medium using dialysis method. The purified sample was then freeze dried, which generated white powder of RSV-PEG conjugate. In the second step, a TOCNFs suspension (0.10 g, 1.0 wt%) was diluted with distilled water, and NHS (0.023 mg, 0.20 mmol) was added to the suspension. After activating for 30 min, the RSV-PEG conjugate (0.22 g, 0.10 mmol) was added to this suspension, and reaction continued for 24 h at 25 °C. The unreacted NHS was removed using dialysis method, and the powder of RPC conjugate was produced via freeze drying. The successful synthesis of RPC conjugate was confirmed by fourier transform infrared spectrometer (FTIR, IFS66V/S, Bruker).

### Preparation of hydrogels

The semi-interpenetrating (semi-IPN) RPC/PB hydrogels were prepared as follows. First, 10.0 g of PVA was added to the RPC suspension (0.8 wt%, 100 g), stirred for 2 h at 90℃. Subsequently, the borax aqueous solution (0.1 g/mL, 10 mL) was added into the aforementioned system, and stirred for 30 min at 90 °C. A stable hydrogel was formed when the solution was cooled to room temperature. A series of hydrogels containing 0.2 wt%, 0.5 wt% and 0.8 wt% of RPC conjugate were prepared and labeled as RPC/PB-0.2, RPC/PB-0.5 and RPC/PB-0.8, respectively. The preparation process of C/PB hydrogel was similar to that of RPC/PB hydrogel, except that RPC conjugate was replaced by CNFs. The PB hydrogel was prepared by adding borax to the PVA aqueous solution.

### Characterization of hydrogels

#### Morphological observation

A scanning electron microscope (SEM, SU8010, Hitachi, Japan) was used to observe the morphology of RPC/PB hydrogels with various RPC contents.

#### Mechanical performance observation

The mechanical properties of the hydrogels were assessed using a universal testing machine (ETM520C, WANCE, China). Hydrogels was cut into uniform rectangular specimens with the size of 10 × 1 × 0.3 cm, and the tensile test were carried with a constant stretching rate of 100 mm/min.

#### Rheological observation

A rheometer (HAAKE MARS III, Thermo Scientific, Germany) was used to examine the rheological behavior of RPC/PB hydrogels. Strain-dependent rheology tests were conducted with the strain swept from 1 to 200% at an angular frequency of 10 rad/s. Also, recovery rest analysis was carried out by straining sample to failure with increasing sweep strain from 1 to 200%, and then using time sweep module to record the recovery of storage modulus (G') and loss modulus (G'').

#### Self-healing experiments

For macroscopic self-healing experiment, the hydrogel samples were sliced in half, and the two halves were brought into contact at 25 ℃ to heal for 10 or 30 min. After that, the tensile test was conducted to evaluate the self-healing capabilities of the RPC/PB hydrogels. In order to verify the role of borate ester bond and hydrogen bond in the hydrogel repair process, the parts of the fractured hydrogel were immersed for 10 min with glucose solution or urea (1 M) before repair, and then tensile test was conducted after splicing to evaluate the repair efficiency.

#### Adhesion measurement

The adhesive strengths of the RPC/PB hydrogels on a variety of substrates were investigated via a lap shear testing method. The substrates were attached to steel plates using a cyanoacrylate glue. Next, hydrogels were placed between two substrates and compressed for 5 min. Then, the adhered plates were pulled to separation at a speed of 10 mm/min on the universal testing machine. The adhesion strength was calculated by the measured maximum load divided by the adhesive areas.

#### Swelling behavior

In this set of analysis, a cylindrical RPC/PB hydrogel sample (diameter = 10 mm, weight = 2 g) was completely immersed in a PBS (0.1 M, pH = 7.4). After reaching swelling equilibrium at room temperature, the surface of the samples was wiped with a filter paper and then dried in vacuum dryer. The swelling ratio (SR) of the samples was determined using the following Eq. 1:

1 $${\text{SR}}\, = \,\left( {{\text{W}}_{{\text{S}}} - {\text{W}}_{{\text{d}}} } \right)/{\text{ W}}_{{\text{d}}}$$

where W_s_ represents the weight of swelling hydrogel and W_d_ represents the dry hydrogel, respectively.

#### Water vapor permeability test

Water vapor permeability (WVP) of RPC/PB hydrogels was gravimetrically determined at 25 °C. Firstly, the RPC/PB hydrogels were made into film shapes with the settled thickness, and then sealed on glass vials using silicone grease. The glass vials had an opening hole (diameter = 2 cm) and of contained 10 mL distilled water, and the vials were placed in a desiccator containing silica gel at 25 °C for 7 h. The exposed hydrogel film region allows water vapor to pass through. The weights of water in the glass vials covered by RPC/PB hydrogels, Tegaderm film or control (i.e., glass vial with no cover) were measured by a four-digit balance. The water vapor permeability (WVP) was calculated according to Eq. 2:

2 $${\text{WVP}}\, = \,\left( {{\text{S}}\, \times \,{\text{d}}} \right)/{\text{A}}$$

where S represents the ratio of the weight loss versus the time, d represents the thickness of hydrogel film, and A represents the area of exposed film.

### In vitro RSV release

To determine the release behavior of RSV from RPC/PB hydrogel, the hydrogel (100 mg) was soaked with 100 mL PBS (0.1 M) solution at various pH levels (pH 5.4, 6.2 and 7.4) in the beaker at 25℃. At determined time intervals, 1 mL of samples were taken from the released solution containing the hydrogels, while 1 mL of fresh buffer was added to keep the system's volume constant. The amounts of RSV in the PBS media were measured using high performance liquid chromatography (HPLC, Agilent 1100, USA). The samples (20 μL) were analyzed by the HPLC with a μ-Bondapak C18 column with 300 mm × 3.9 mm; the mobile phase of acetonitrile/water (40/60, V/V); flow rate of 0.60 mL/min; UV detector at the wavelength of 306 nm and 280 nm at room temperature. The cumulative release of RSV was calculated as follows:

3 $${\text{Er }}\left( \% \right) \, = \frac{{V_{e} \sum\nolimits_{1}^{n - 1} {C_{i} } + V_{0} C_{n} }}{{m_{0} }} \times 100\%$$

Where Er: cumulative drug release efficiency; V_e_: displacement volume of PBS; V_0_: total volume of release medium; C_i_: concentration of the released fluid at the displacement sampling; m_0_: the total weight of the drug contained in the hydrogels; n: number of times to replace PBS.

### In vitro cytocompatibility

To evaluate the cytocompatibility, dried C/PB and RPC/PB hydrogels were ground to powder and cultured with L929 cells (the density of cells was 2 × 10^7^/mL) for 24 h at 37 °C. Then cells were dyed with MTT, and the optical density of the system was measured at 570 nm by a microplate reader (Tecan, Infinite 200 PRO, Switzerland).

The hemocompatibility of C/PB and RPC/PB hydrogels was determined by a hemolysis assay according to a previous report [58]. First, the hydrogels were soaked in diluted mouse blood for 1 h at 37℃. Subsequently, the supernatants of blood samples were collected after centrifugation, and the absorbance of the supernatants was measured by a microplate reader at 540 nm. Diluted mouse blood in PBS (0.1 M, pH 7.4) and Triton X-100 were used as negative control and positive control, respectively.

### In vitro antibacterial assay

The antibacterial performance of PB, C/PB and RPC/PB hydrogels were evaluated in vitro using *S. aureus* as bacteria models through the inhibition zone method [59–61]. First, the hydrogels were sliced into discs of 20 mm in diameter, and each side of the disked hydrogels was treated with UV light for 30 min. Then, *S. aureus* in LB broth (10^6^ CFU/mL, 100 μL) was transfer to the agar culture medium and spread evenly. After that, the sterilized hydrogel disks were placed in the center of the bacteria-bearing medium plate. After incubated for 24 h, the inhibition zone around the samples were observed, and their diameters were measured.

The antibacterial activity of the samples was quantitatively determined by the spread plate method [62]. In 96-well plates, *S. aureus* stock suspensions in LB broth (106 CFU/mL) and hydrogel samples were added and incubated for 1 h at 37 °C. After diluted for 1000 times, the mixture solutions were evenly deposited on LB agar plates and incubated at 37 °C for 24 h. The amounts of CFUs on the LB agar plates was manually counted. Equation 4 was used to calculate the bacterial survival ratio:

4 $${\text{Survival ratio }}\left( \% \right)\, = \,{\text{CFU}}/{\text{CFU}}_{0} \, \times \,{1}00\%$$

where CFU and CFU_0_ represent the amounts of colonies formed with or without hydrogels, respectively.

### In vitro antioxidant assay

To evaluate the antioxidant activity of RPC/PB hydrogels, 40 mg of hydrogel samples were mixed with DPPH• ethanol solution (100 μM, 3 mL) for 30 min in a dark environment. Next, the mixture solution was centrifuged, and the absorbance of DPPH• in supernatant was tested by a UV–Vis spectrophotometer (Agilent 8454, USA). The scavenge ratio of DPPH• was determined following Eq. 5:

5 $${\text{DPPH}} \cdot {\text{scavenging }}\% \, = \,\left( {{\text{A}}_{{\text{B}}} - {\text{A}}_{{\text{H}}} } \right)/{\text{A}}_{{\text{B}}} \, \times \,100\%$$

Where A_B_ represent the blank group (DPPH• + ethanol), and A_H_ represent hydrogel groups (DPPH• + ethanol + hydrogels).

### In vivo wound healing assay

Female KM mice were randomly divided into three groups (n = 6): control, C/PB-0.5 and RPC/PB-0.5 hydrogel group. A partial thickness wounds with a 6 mm diameter were made on the dorsum of each mouse under anesthesia. The dried C/PB-0.5 hydrogel was soaked in 200 µL of *S. aureus* (10^6^ CFU/mL) for 45 min, and the wounds of all mice were covered with soaked C/PB-0.5 hydrogel to infect *S. aureus*. After that, the wound areas of the C/PB-0.5 group were completely covered with C/PB-0.5 hydrogel dressings, the RPC/PB-0.5 group was covered with RPC/PB-0.5 hydrogel dressings, respectively, and secured with Tegaderm. There was no further treatment for the control group. The hydrogel dressings were replaced every three days, and the images of the wounds were recorded with a digital camera on days 0, 3, 6, 9, and 12.

### Histological analysis of wounds

To evaluate the tissue regeneration of wound, the skin tissue samples from wound site were taken, fixed with 4% paraformaldehyde and embedded with paraffin to obtain the tissue slides. After staining with hematoxylin and eosin (H&E) and Masson’s trichrome, the histological images of samples were observed using an optical microscope (IX53, Olympus, Japan). For the biochemical analysis, the tissue samples were collected after 12 d treatment and made into disc-shaped tissue, and its collagen amount was evaluated by estimating hydroxylproline content using a commercial kit [63]. Additionally, the major organs, including the heart, liver, spleen, lungs, and kidneys, were collected and stained with H&E for histological analysis.

## Results and discussion

### Synthesis of RPC conjugate and preparation of RPC/PB hydrogel

**Scheme 1:**
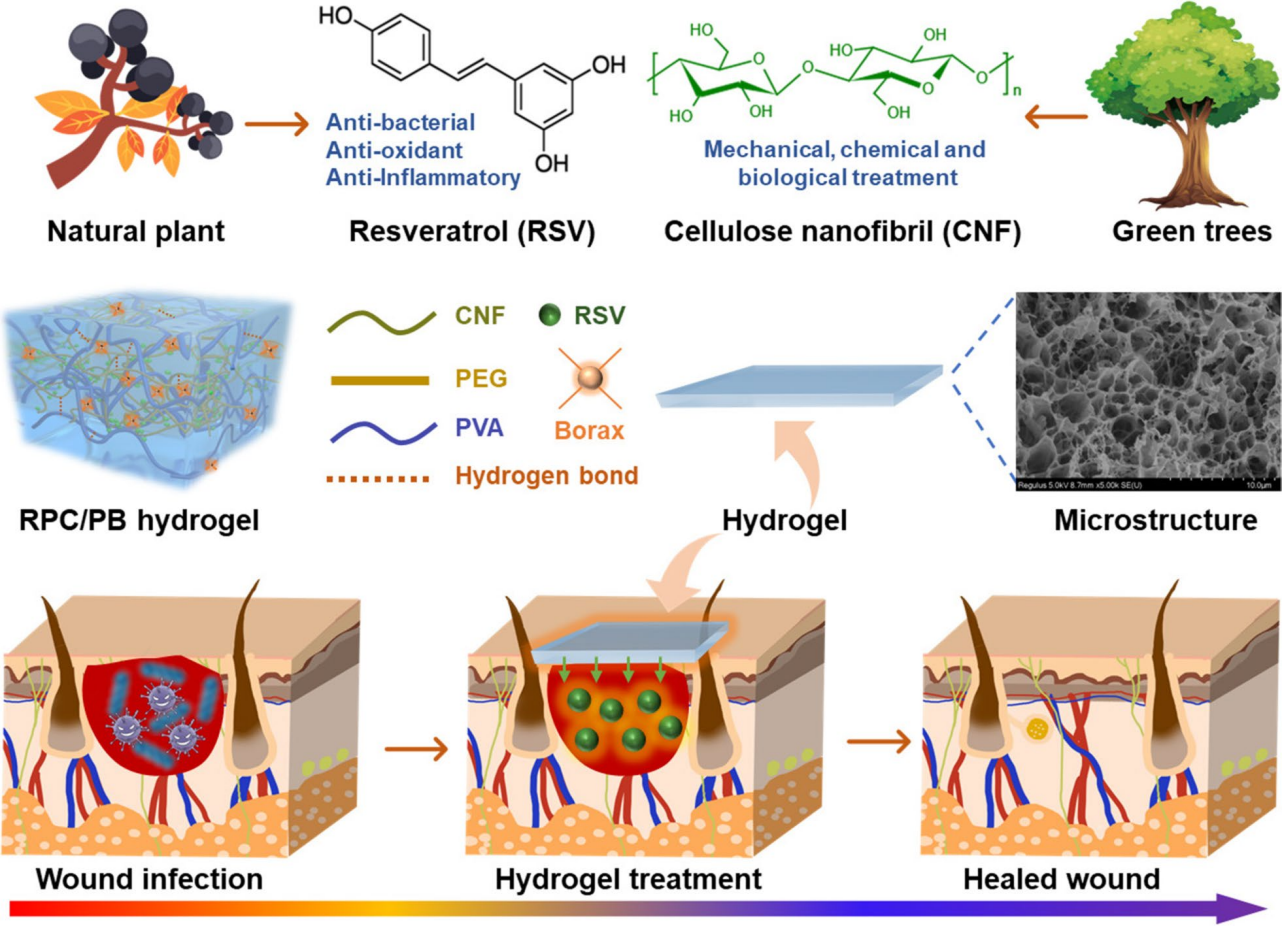
Schematic and architecture of the RPC/PB hydrogel and the application of the hydrogel in wound healing

**Fig. 1:**
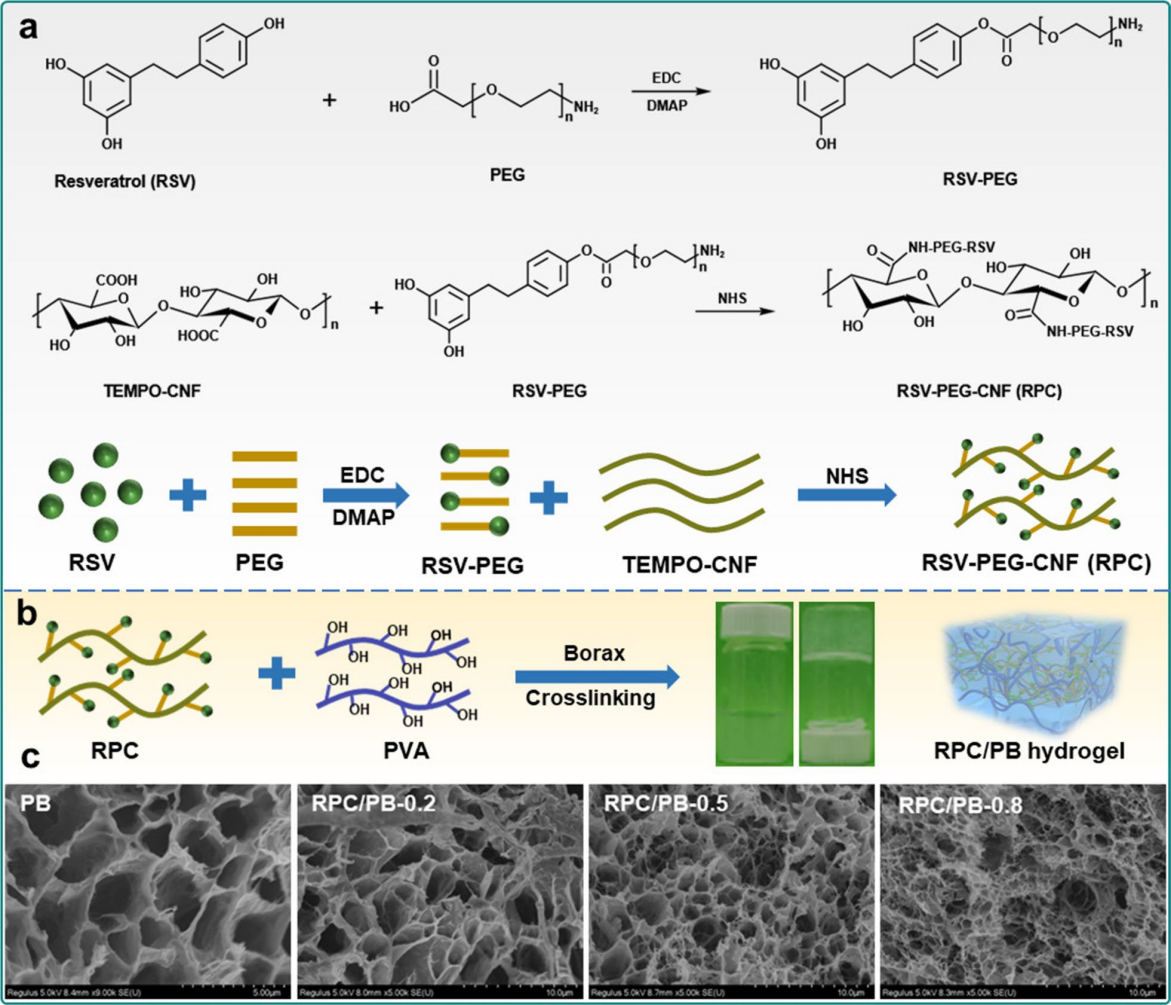
**a** Synthetic route of RPC conjugate. **b** Schematic representation of RPC/PB hydrogel preparation. **c** SEM images of PB hydrogel and RPC/PB hydrogels with different RPC content

TEMPO-oxidized CNFs and PVA are employed as skeleton to fabricate hydrogels, which have the distinct individual and complementary features of PVA and CNFs (Scheme 1). In this hydrogel matrix, PVA was selected as the primary polymer network structure due to its high elasticity and good biocompatibility, and CNFs were chosen as nanofillers to further enhance the mechanical properties of the hydrogel [64–66]. Additionally, to endow hydrogel with good antibacterial and antioxidant properties, CNFs was grafted with natural antibiotic RSV using PEG as linker before the hydrogel preparation. The synthesis route of RPC conjugate is illustrated in Fig. 1a. First, RSV was reacted with PEG via esterification, and then the obtained RSV-PEG was reacted with CNFs through amidation to synthesize RPC conjugate. The successful synthesis of RPC conjugate was proved by FTIR analysis. As shown in Additional file 1: Fig. S1, the peaks at 3290 cm^−1^ and 1650 cm^−1^ in PEG belonged to the N–H and C = O, respectively [67]. After reacting with RSV and CNFs, the peaks at 1768 cm^−1^ (C = O stretch in ester), 1687 cm^−1^ (C = O stretch in ester), 1640 cm^−1^ (-NH bending) and 1205 cm^−1^ (C-O stretching vibration in ester) were appeared in RPC conjugate, which demonstrated the successful synthesis of RPC conjugate [68]. The gelation of the RPC/PB hydrogel was shown in Fig. 1b. In the hydrogel matrix, the PVA chains are crosslinked with borax through dynamic borate bonds to form a hydrogel network, and the RPC conjugate was interpenetrated in the PVA/borax network to form a semi-interpenetrating network to reinforce the mechanical strength of the hydrogel. Meanwhile, the multiple hydrogen bonds were formed in the RPC-PVA, PVA-PVA and RPC-RPC chains to further promote the RPC/PB hydrogel formation. These interactions work together to give the RPC/PB hydrogel with unique physicochemical features.

The morphology of PB, RPC/PB-0.2, RPC/PB-0.5 and RPC/PB-0.8 hydrogels were observed by SEM. As shown in Fig. 1c, all hydrogels showed porous structure. Compared with PB hydrogel, RPC/PB hydrogels displayed more denser structure, and the structure density increased with the increase of RPC content. The reason for this phenomenon might be that RPC and PVA can form interwoven networks, leading to the decrease of the space between the PVA chains. The hydrogels' intrinsic interwoven porous structure can significantly improve wound healing, as the porous structure allows blood absorption and water vapor permeation [69].

### Mechanical, rheology and self-healing properties of hydrogel

Generally, a compact structure of hydrogels produces robust mechanical properties. Thus, we hope that the mechanical strength of hydrogel could be enhanced through the reinforcing effect of CNFs. The mechanical strength of hydrogel was determined and shown in Table 1. The PB hydrogel exhibited unsatisfied mechanical performance with 582% strain at break and 32.4 kPa ultimate stress. Excitingly, the ultimate stress of RPC/PB hydrogel was significantly increased, which is up to 117.4, 149.6, and 138.7 kPa when 0.2 wt%, 0.5 wt% and 0.8 wt% of RPC were applied to the hydrogel, respectively. Likewise, when the RPC content increased, the Young's modulus of RPC/PB hydrogel increased as well, reaching 107.6 kPa at 0.5 wt% RPC content, which is 3.89 times that of PB hydrogel. Surprisingly, the strain also increased, reaching 1246% at 0.5 wt% RPC content, which is 2.14 times as much as that of PB hydrogel. This result demonstrated that the prepared RPC/PB hydrogel is stronger and tougher compared with PB hydrogel. Moreover, the strain at break value of RPC/PB hydrogel decreased when the RPC content is increased to 0.8 wt%, which is common characteristic of nanofillers reinforced polymer composites [70]. At a higher RPC concentration, the excess RPC would aggregate, leading to unequal stress distribution in the hydrogel [71]. Even so, the strain and stress can still reach 1184% and 138.7 kPa at 0.8 wt% RPC content, suggesting satisfying elastic performance. Meanwhile, the toughness of the RPC/PB hydrogel was also increased compared with PB hydrogel. This excellent mechanical performance is attributed to the reinforcing effect of RPC, which can form hydrogen bonds and/or physical entanglement with PVA chains as well as with themselves.

**Table 1:** Mechanical properties of hydrogels

Samples	Fracture strain (%)	Fracture strength (kPa)	Young's modulus (kPa)	Toughness (MJ/m.^3^)
PB	582	32.4	27.6	0.37
RPC/PB-0.2	1048	117.4	94.8	0.84
RPC/PB-0.5	1246	149.6	107.6	1.47
RPC/PB-0.8	1184	138.7	114.9	1.39

**Fig. 2:**
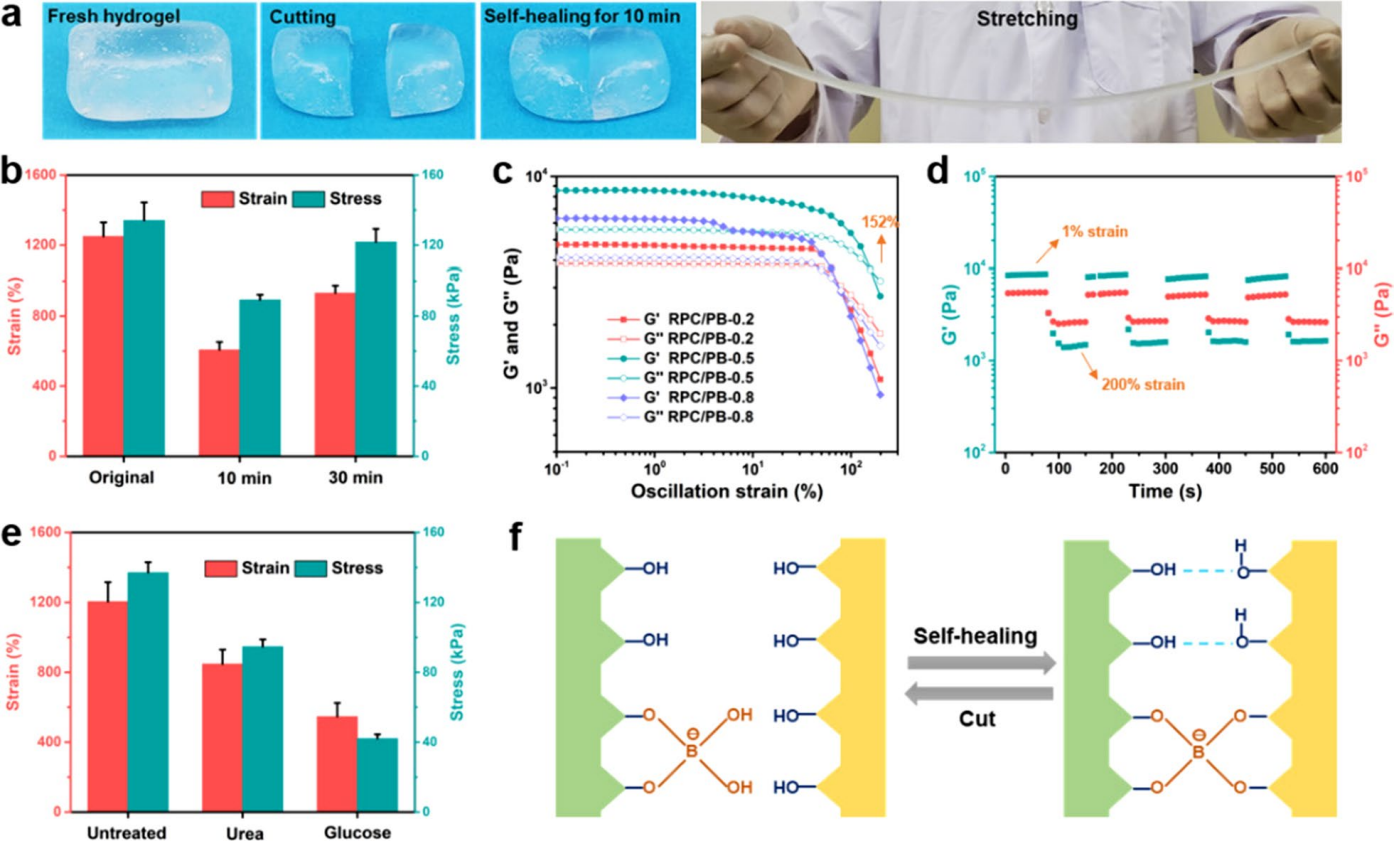
**a** Display of the macroscopic self-healing performance of RPC/PB-0.5 hydrogel. **b** Repair efficiency of RPC/PB-0.5 hydrogel. **c** Strain-dependent rheology measurement of the RPC/PB hydrogels with the strain sweeping from 1 to 200% at an angular frequency of 10 rad/s. **d** Tixotropic experiment during the cyclic strain changes between 1% strain and 200% strain of RPC/PB-0.5 hydrogel. **e** Influences of urea and glucose on repair efficiency of RPC/PB-0.5 hydrogel. **f** Schematic diagram of hydrogel repair mechanism

The self-healing performance of the RPC/PB hydrogel was analyzed to evaluate its ability to withstand external damage. The macroscopic self-healing ability was first evaluated using RPC/PB-0.5 as model hydrogel. From Fig. 2a, we can see that the severed hydrogel fragment can be restored to a complete hydrogel without external intervention, and the healed hydrogel can be stretched to more than 10 times than its original length. To evaluate the self-healing efficiency, we next investigated the tensile properties of RPC/PB-0.5 hydrogel at different healing times. As shown in Fig. 2b, the fracture stress of the healed hydrogel increased as the healing time extended. After healing for 30 min, the healing efficiency reached to 90.7%, suggesting the excellent self-healing performance of RPC/PB-0.5 hydrogel.

The rheological test was further investigated to demonstrate the RPC/PB hydrogel's self-healing capacity. We first analyzed the strain amplitude sweep of the RPC/PB hydrogel. As shown in Fig. 2c, the G′ curve and G″ curve of RPC/PB-0.5 crossed at the strain of 152%, showing that the hydrogel structure would be entirely disrupted when the strain was greater than 152%. The rheological recovery behavior of the hydrogel was then assessed using a continuous strain cycle change from 1 to 200% with 70 s between each step (Fig. 2d). It can be seen that the hydrogel structure crumbled when exposed to larger strain (200%), yet the hydrogel was able to repair its form at a smaller strain (1%), revealing the RPC/PB hydrogel's exceptional self-healing capability.

The macroscopic self-healing test and rheological recovery test demonstrated the excellent self-healing capability of the RPC/PB hydrogel. In order to clarify the role of borate ester and hydrogen bonds in hydrogel repair, the freshly cut hydrogel pieces were treated with urea or glucose solution before hydrogel repair. As shown in Fig. 2e, the fracture stress of the healed RPC/PB-0.5 hydrogel decreased to 43.2 kPa after glucose treatment, which is only 30.7% of the original hydrogel. According to previous studies, the diol groups of glucose are easily form complexes with borate ions, which seriously impede the rebuilding of borate ester bonds at the fracture interfaces of the hydrogel [72, 73]. However, urea treatment had slightly effect on the fracture stress of hydrogel, which was still up to 97.1 kPa, despite the fact that urea could destroy the hydrogen bond at the fracture surfaces [74, 75]. These findings suggested that, in comparison to hydrogen bond, the dynamic borate ester bond is more critical in the hydrogel’s self-healing process.

### Adhesion, swelling ratio and water vapor permeability test of hydrogels

**Fig. 3:**
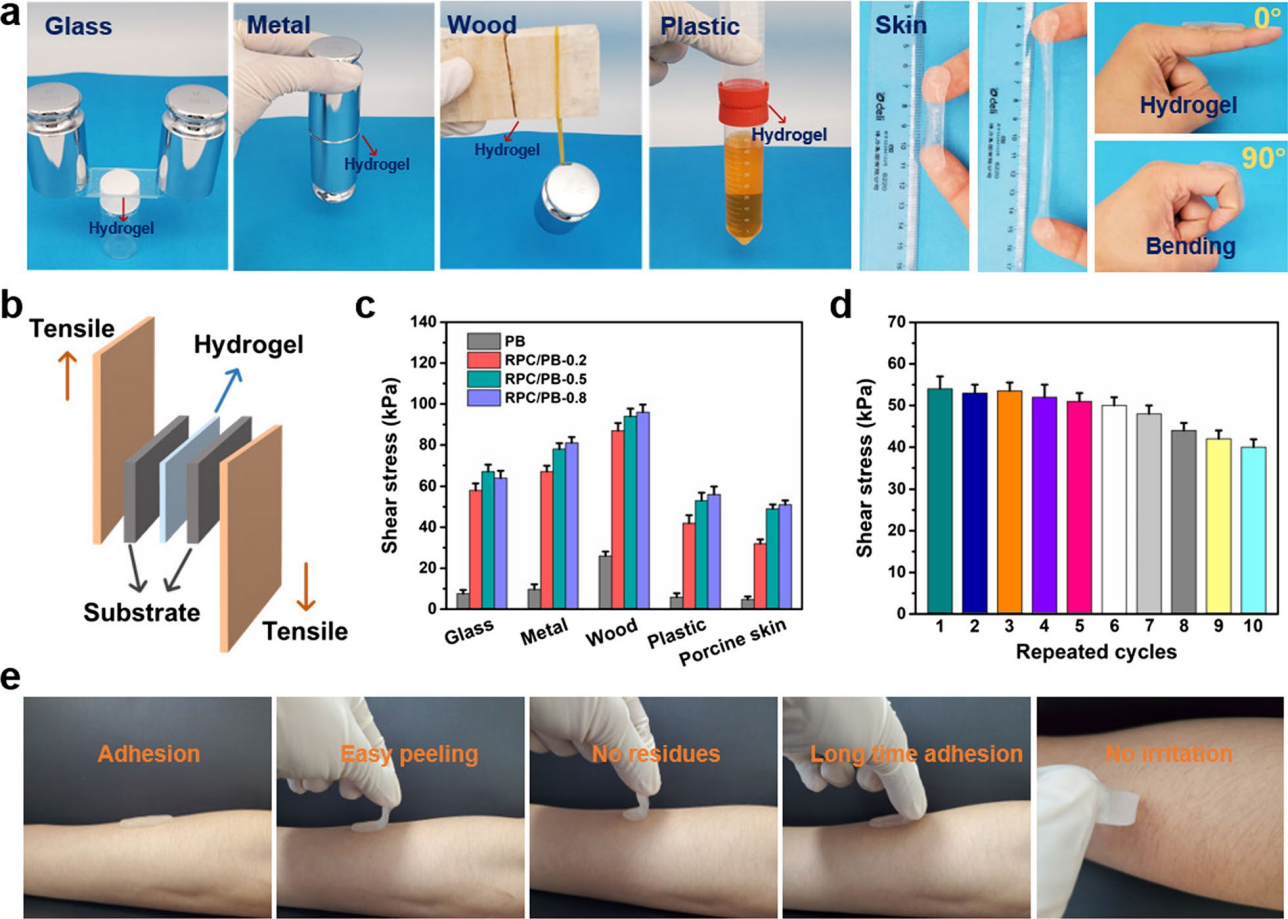
**a** The RPC/PB-0.5 hydrogel firmly adhered to diverse surfaces and moved with the finger joint. **b** Schematic representation of the adhesion measurements of the hydrogels. **c** Adhesive strength of the PB and RPC/PB hydrogels to various substrates. **d** Repeatable adhesion properties of the RPC/PB-0.5 hydrogel on porcine skin. **e** The RPC/PB-0.5 hydrogel is removed from human skin with no residue

Adhesion allows the wound dressings to be in a close contact with the skin surface. Thus, we examined the adhesion performance of the PRC/PB hydrogel. From Fig. 3a, we can see that the RPC/PB-0.5 hydrogel showed good adhesion properties to various substrates, such as wood, metal, plastic and glass. Particularly, such hydrogel displayed strong tissue-adhesive activity, allowing them to be directly attached to human skin and tolerate stretching or moving with the finger joint. A tensile adhesive test was designed to quantify the adhesive strength of the PB and RPC/PB hydrogel toward various surfaces (Fig. 3b). The RPC/PB-0.5 hydrogel showed a significant increase in adhesive strength when compared to the PB hydrogel, as seen in Fig. 3c, which was evidently impacted by diverse substrates. Significantly, the RPC/PB-0.5 hydrogel exhibited durable and repeatable adhesive behavior on porcine skin owing to the reversibility of the physical interactions. As displayed in Fig. 3d, there was slightly decrease in adhesive strength after 10 detachment-reattachment cycles, indicating the hydrogel's high reusability. Furthermore, the hydrogel adhered well to human skin, and did not harm the skin after long-term adhesion (Fig. 3e).

The swelling behavior of PB hydrogel and RPC/PB hydrogels was measured by soaking hydrogel in the PBS at room temperature [76]. The swelling ratio with different soaking times was shown in Additional file 1: Fig. S2. After soaking for 42 h, both PB and RPC/PB hydrogels reached the swelling equilibrium state. The swelling ratio of RPC/PB hydrogels was significantly lower in comparation with the PB hydrogel, and the RPC/PB-0.8 with highest content of RPC showed the lowest swelling ratio. The decrease in the swelling rate may be attributed to the intensified crosslinking density of RPC/PB hydrogel [77].

Suitable water vapor permeability (WVP) of the dressing is critical for wound healing. According to previous studies, we know that the optimal WVP of wound dressings should be 2000–2500 g/m^2^ per day, which could keep the balance between the water losses and surface moisture at the wound site [78]. As shown in Additional file 1: Fig. S3, the commercial Tegaderm film showed relatively low water vapor permeability, which was 1162 g/m^2^ per day. On the contrary, all the RPC/PB hydrogels displayed a higher water vapor permeability, reaching 2419, 2047, and 1758 g/m^2^ per day for RPC/PB-0.2, RPC/PB-0.5, and RPC/PB-0.8, respectively. This suitable water vapor permeability of RPC/PB hydrogels could maintain the balance between water vapor diffusion and water absorption, balancing fluids on the wound site.

### In vitro RSV release, biocompatibility and antioxidant activity of hydrogel

**Fig. 4:**
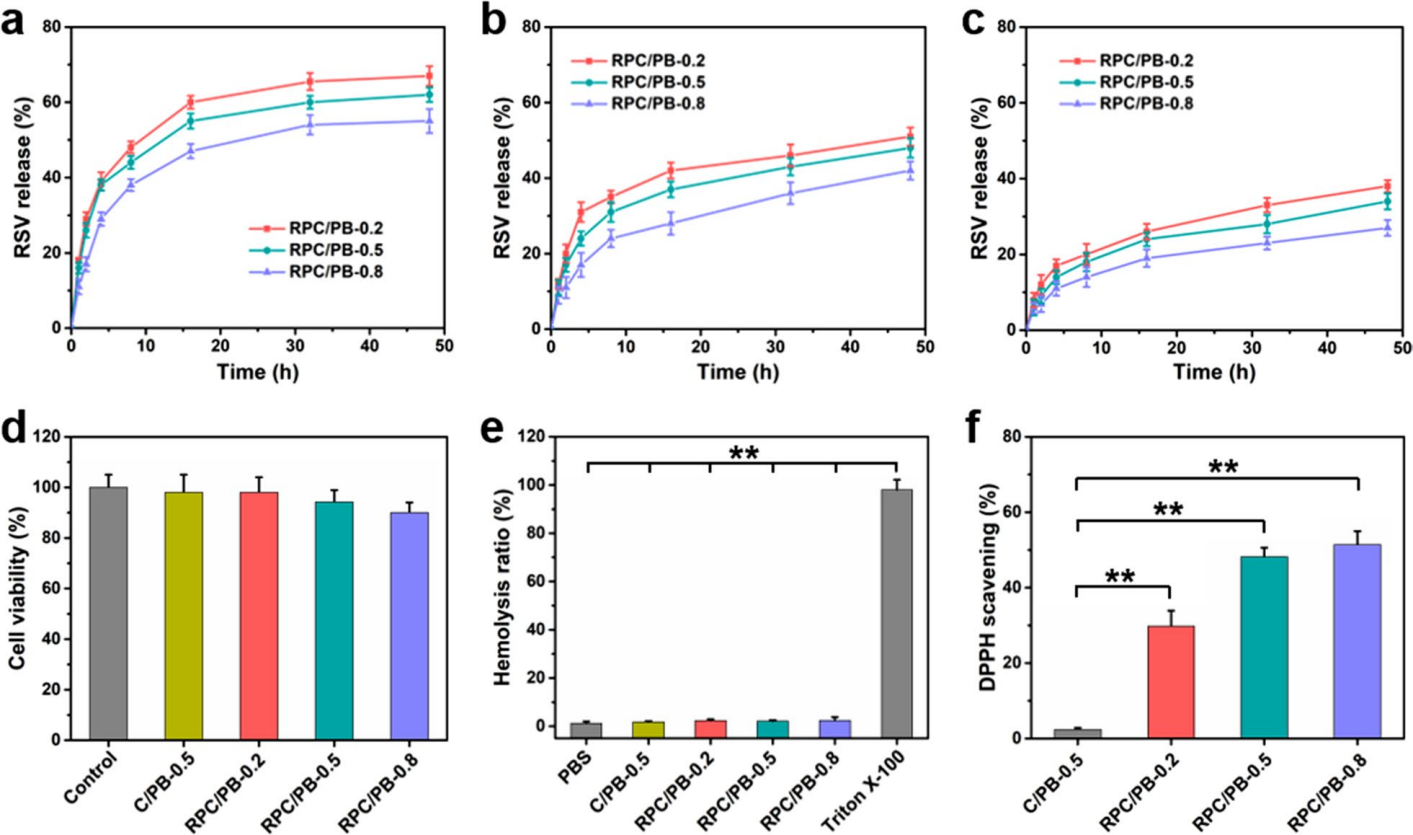
**a**–**c** In vitro release profiles of RSV from the RPC/PB hydrogels at different pH values (pH 5.4, 6.2 and 7.4). **d** In vitro cytotoxicity of C/PB-0.5 and RPC/PB hydrogels against L929 cells. **e** In vitro hemocompatibility analysis of C/PB-0.5 and RPC/PB hydrogels. **f** DPPH scavenging percentage by C/PB-0.5 and RPC/PB hydrogels. **p < 0.01

Various studies have reported the modulating effect of RSV on wound healing due to its excellent anti-bacterial, anti-oxidant and anti-inflammatory activity [79, 80]. Thus, we selected RSV as bioactive agent and encapsulated it in the hydrogel matrix to promote wound healing. Additionally, we known that the majority of wound types, including acute, chronic and suppurative wounds, show an acidic pH level. And thus, it is desirable to develop novel wound dressings with pH-responsive drug release behavior. The in vitro drug release behavior of the RPC/PB hydrogels was investigated in different pH values (pH = 5.4, 6.2 and 7.4). As shown in Fig. 4a–c, RPC/PB hydrogels released the drug faster in acidic environment (pH 5.4 and 6.2) than normal physiological environment (pH 7.4). Specifically, the RPC/PB hydrogels exhibited a greater release rate in more acidic environment (pH 5.4 > 6.2 > 7.4) in the earlier release stage. After 48 h of incubation, approximately 64.2% of RSV was released from RPC/PB-0.5 at pH 5.4, whereas about 48.4% and 27.6% RSV were released at pH 6.2 and 7.4. This result showed that such RPC/PB hydrogels with pH-responsive can achieve controlled release of drug, which was adapted to the most of wounds and physiological skin environment. This pH-responsive release property of the hydrogels might be ascribed for the pH-dependent degradation behavior of RPC/PB hydrogels. Moreover, the effect of cross-linking density on the release behavior was also investigated in PBS under the same pH value. As shown in Fig. 4a–c, the drug release rate decreased with the increase of RPC content in both pH 5.4, 6.2 and 7.4. This phenomenon might be attributed to that RPC/PB hydrogel with high RPC content exhibited higher structural density, which is not convenient for drug diffusion compared with other hydrogel formulations with low density.

Good biocompatibility is one of the most important factors of wound dressing. Thus, we next investigate the biocompatibility of the produced hydrogels. The cytocompatibility of the C/PB-0.5 and RPC/PB hydrogels was tested by a standard MTT assay [81]. It can be seen that there was no obvious cytotoxicity of hydrogels, more than 90% of cells were viable (Fig. 4d). A hemolysis assay was used to investigate the blood compatibility of the C/PB-0.5 and RPC/PB hydrogels. As depicted in Fig. 4e, no evident hemolysis was found in both C/PB-0.5 and RPC/PB hydrogel groups. Moreover, the RPC/PB hydrogels with different RPC content (RPC/PB-0.2, RPC/PB-0.5 and RPC/PB-0.8) did not show significant differences in cytocompatibility and hemocompatibility. These results revealed that the produced RPC/PB hydrogels safe enough for biomedical application.

Large amounts of free radicals are known to reside in the wound site, and these radicals would cause oxidative stress and enzyme inactivation, seriously hindering the healing process of wounds [82]. It has been reported that RSV can effectively scavenge free radicals by a variety of mechanisms. Herein, the scavenging efficiency for DPPH• was used to assess the antioxidant activity of RPC/PB hydrogels. From Fig. 4f, we can see that the intensity of the DPPH• absorption peak significantly decreased after incubating with RPC/PB hydrogels, suggesting the excellent antioxidant activity of these hydrogels. Furthermore, when the amount of RSV in the hydrogel increased, so did the antioxidant activity. In contrast, hydrogel without RSV (C/PB-0.5) showed a weak antioxidant activity.

### Antibacterial activity of hydrogel

**Fig. 5:**
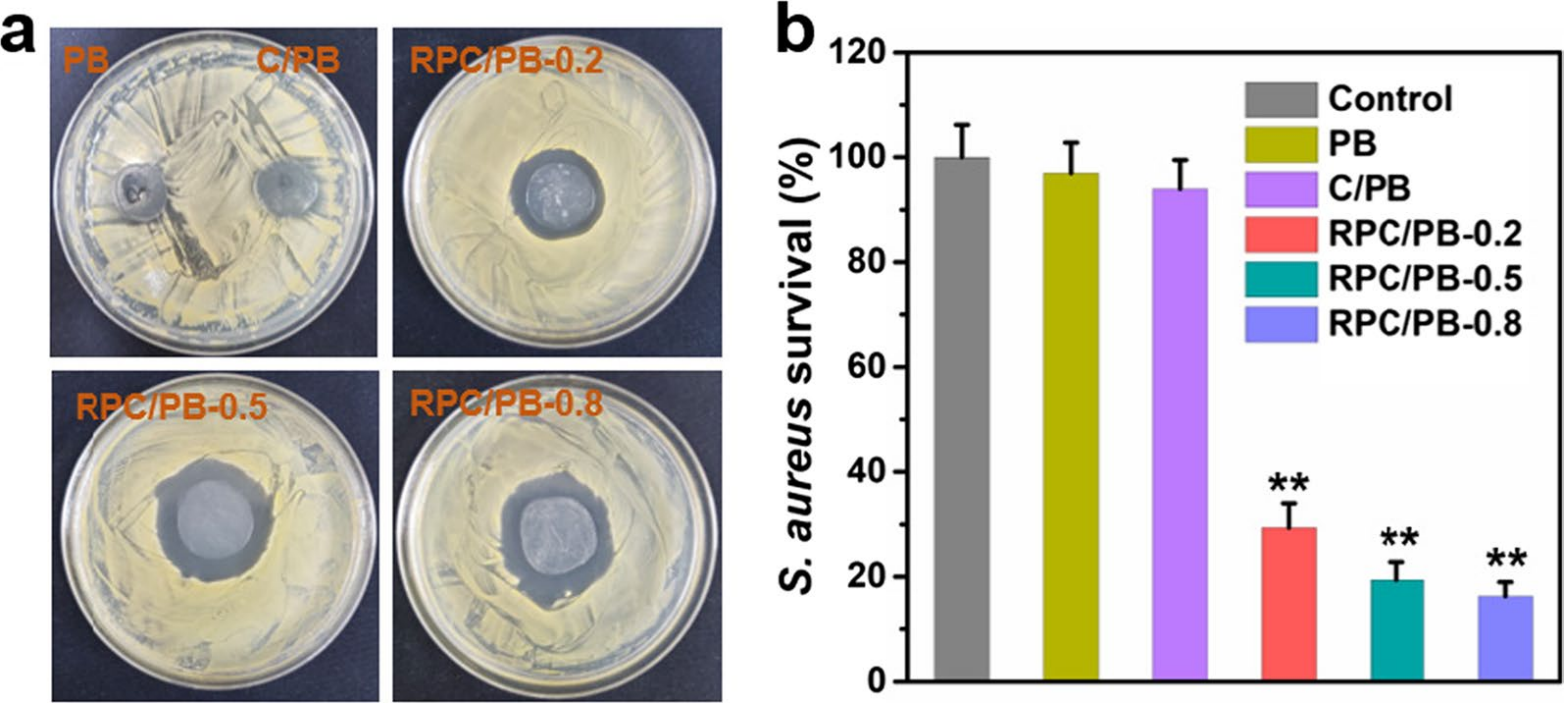
In vitro antibacterial activities of the PB, C/PB-0.5 and RPC/PB hydrogels against *S. aureus*. **a** Pictures of inhibition zone and **b** corresponding statistical data of colonies of *S. aureus*. **p < 0.01

The antibacterial activity of the hydrogels was investigated in vitro using *S. aureus* as a model bacterial, which would be associated with the majority of wound infections [83]. The inhibition zone method was employed to investigate the antibacterial activity of the hydrogel. As depicted in Fig. 5a, the inhibition zone around RPC/PB hydrogels were clear, while PB and C/PB hydrogel showed no inhibition zone, suggestion the potent antibacterial activity of RPC/PB hydrogel. A spread plate method was further performed to assess the antibacterial activity of the hydrogels. As shown in Fig. 5b, the PB and C/PB groups exhibited weak antibacterial ability, which had no significant difference with the control samples. In contrast, RPC/PB hydrogel killed most of *S. aureus*. In this case, we observed 29.4%, 19.2% and 15.7% survival ratios for RPC/PB-0.2, RPC/PB-0.5 and RPC/PB-0.8, respectively. The above results indicated that RPC/PB hydrogel has potent antibacterial ability against *S. aureus*, and the antibacterial effect increased with increasing RPC content.

### In vivo wound healing assay

**Fig. 6:**
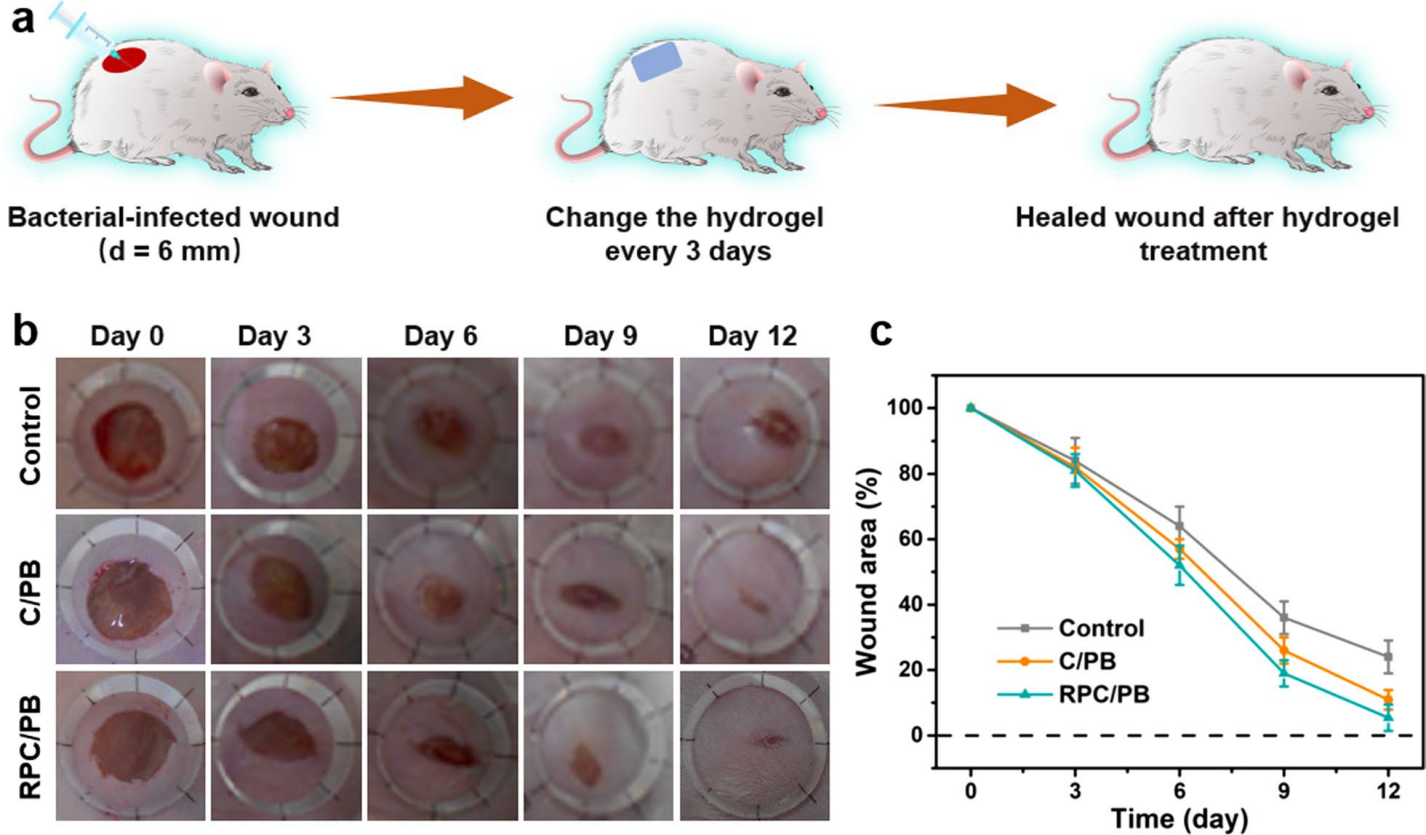
In vivo evaluation of hydrogels in *S. aureus*-infected wound healing. **a** Schematic illustration of creating *S. aureus*-infected mice wound model. **b** Pictures of the wound area on the 3rd day, 6th day, 9th day and 12th day for control, C/PB-0.5 hydrogel and RPC/PB-0.5 hydrogel groups. **c** Changes in wound size for control, C/PB-0.5 hydrogel and RPC/PB-0.5 hydrogel groups

A bacterial-infected wound model was established to evaluate the wound healing effect of the RPC/PB hydrogel (Fig. 6a). Following hydrogel treatments, the wound healing process was recorded with digital camera. As shown in Fig. 6b, the RPC/PB-0.5 hydrogel treated group markedly accelerated the wound closure in comparation with other groups (the control and C/PB-0.5). After 12 days of therapy, wounds treated with RPC/PB-0.5 hydrogel showed no visible open wounds and were smooth with new epidermal tissue, whereas 24.2% and 11.7% of the wounds in the control and C/PB-0.5 groups remained open (Fig. 6c). The results revealed that this RSV-encapsulated RPC/PB-0.5 hydrogel can accelerate the wound closure and skin regeneration.

### Histological and immunohistochemistry examination of skin wounds

**Fig. 7:**
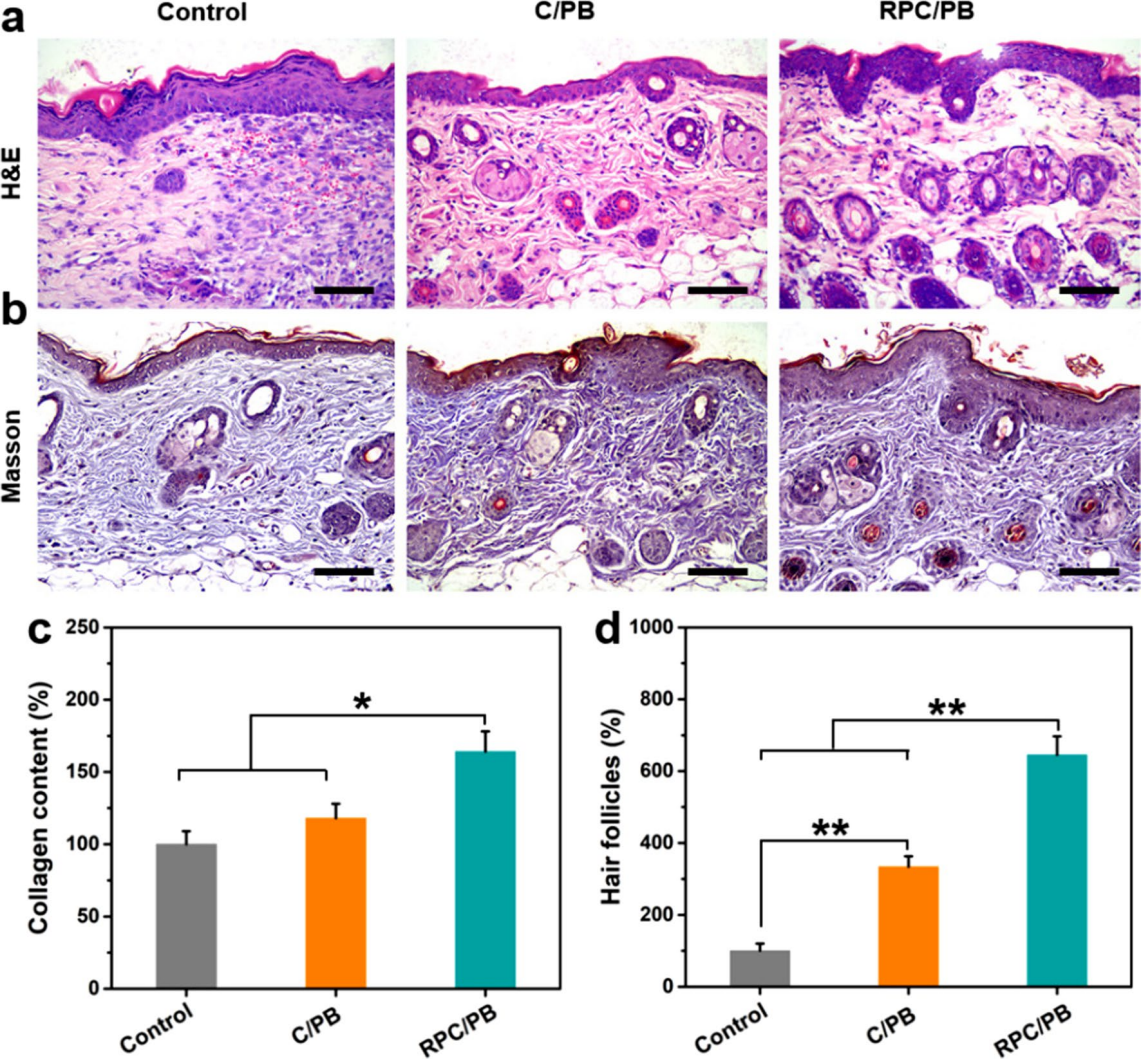
Micrographs of H&E-stained tissue slices **a** and Masson’s trichome-stained tissue slices **b** from control, C/PB-0.5 hydrogel and RPC/PB-0.5 hydrogel groups after 12 days of treatment (scale bar: 200 μm). **c** Collagen amount in control, C/PB-0.5 hydrogel and RPC/PB-0.5 hydrogel groups by determining the hydroxyproline on day 12. **d** Amount of hair follicles in control, C/PB-0.5 hydrogel and RPC/PB-0.5 hydrogel groups on the 12th day, and number in the control group was set as 100%. *p < 0.05 and **p < 0.01

Following the investigation of wound closure with various hydrogels, histological testing was used to further analyze wound healing. After 12 days of treatment, skin tissues from the control, C/PB-0.5 hydrogel and RPC/PB-0.5 hydrogel groups were collected and stained with H&E. As shown in Fig. 7a, the RPC/PB-0.5 hydrogel treated group had almost complete regeneration of dermis tissue with skin appendages such as hair follicles, revealing better tissue regeneration of RPC/PB-0.5 hydrogel. In comparison, the control and the C/PB-0.5 hydrogel groups demonstrated incomplete epidermal and dermal tissue regeneration.

Masson’s trichrome staining was performed to depict the deposition of collagen fibers in the wound healing process. As depicted in Fig. 7b, the collagen deposition was found in both the control, C/PB-0.5 hydrogel and RPC/PB-0.5 hydrogel groups. However, the collagen deposition was denser and more organized in the RPC/PB-0.5 group compared with control and C/PB-0.5 groups. The amount of deposited collagen in the granulation tissue was analyzed by measuring the hydroxyproline content. From Fig. 7c, we can see that the RPC/PB-0.5 hydrogel group exhibited superior collagen deposition at wound site after 12 days treatment compared with control and C/PB-0.5 groups. In addition, the amount of hair follicles was counted at the wound site to further evaluate the wound healing. The number of hair follicles in the control group was set as 100%. Compare with control group, 334% and 645% hair follicles were found in C/PB-0.5 and RPC/PB-0.5 hydrogel group, respectively.

**Fig. 8:**
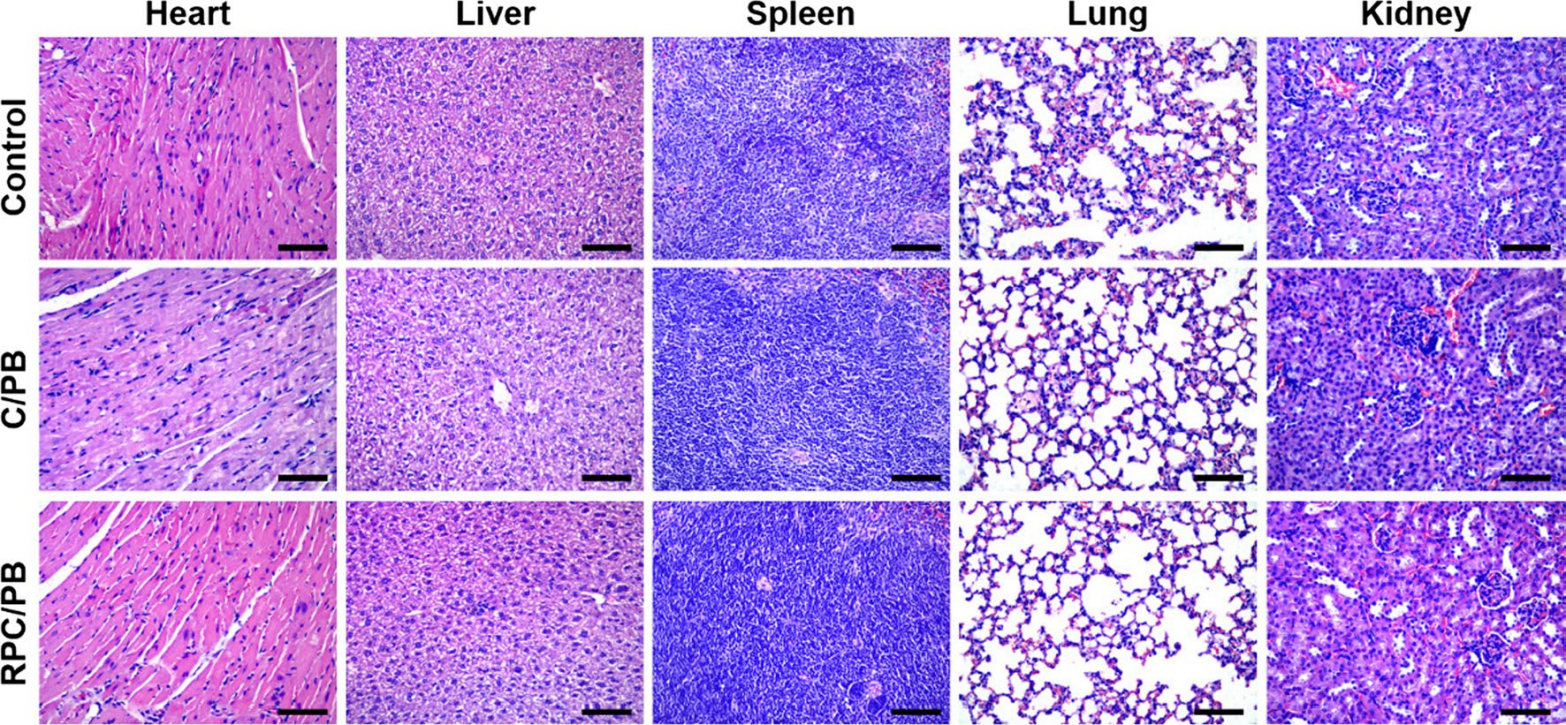
Micrographs of H&E-stained major organ tissue slices from control, C/PB-0.5 hydrogel and RPC/PB-0.5 hydrogel groups after 12 days of treatment (scale bar: 200 μm)

After 12 days of treatment, histological analysis of the major organs (heart, liver, spleen, lungs, and kidneys) showed integrated tissue structure with no aberrant abnormalities (Fig. 8), reflecting the high safety of prepared hydrogel.

## Conclusion

In this work, we reported a novel pH-responsive hydrogel for bacteria-associated wound healing. In this hydrogel matrix, PVA/borax (PB) crosslinked by dynamic borate ester bonds and hydrogen bonds was selected as main network. Also, CNFs interpenetrated in the PB network to enhance the mechanical strength of the hydrogel by promoting extra hydrogen bonds and physical entanglement. To endow the hydrogel with excellent antibacterial and antioxidant ability, the natural antibiotic RSV was grafted to CNFs before the hydrogel preparation. The results showed that the mechanical strength of CNFs-reinforced PB hydrogel reached 149.6 kPa, which was 4.62 times as much as that of the PB based hydrogel. Due to the dynamic borate ester and hydrogen bonds in the hydrogel network, the hydrogel showed high self-healing rate and self-healing efficiency. Importantly, such hydrogel displayed pH-responsive drug release behavior, with cumulative RSV release at pH 5.4 to be 2.33 times that at pH 7.4, which was adapted to the acidic environment of the wounds. Moreover, this hydrogel exhibited potent antioxidant and antibacterial activities due to the introduction of RSV. Take advantage of these properties, accelerated skin regeneration and wound healing processes was achieved. Overall, this hydrogel could be a good alternative to current wound dressings and has great potential in the field of clinical treatment of wound infection.

## Supplementary Information

**Additional file 1: Fig. S1.** FTIR spectra of PEG, RSV, CNF and RSV-PEG-CNF conjugate. **Fig. S2.** Swelling ratio of different hydrogel groups. **Fig. S3.** Water vapor permeability of control, commercial Tegaderm film and RPC/PB hydrogel groups with different RPC content. **Fig. S4**. SEM images of RPC conjugate, PB, RPC/PB-0.2, RPC/PB-0.5 and RPC/PB-0.8 hydrogels. **Fig. S5.** Storage modulus (G') and loss modulus (G'') of PB, RPC/PB-0.2, RPC/PB-0.5 and RPC/PB-0.8 hydrogels versus frequency. **Fig. S6.** RSV release profiles from RPC conjugate under pH 5.4, 6.2 and 7.4. **Fig. S7. **FTIR spectra of PB, C/PB-0.5 and RPC/PB-0.5 hydrogels.

## Data Availability

All data generated or analyzed during this are included in this published article.
