# Supplementary material for: A cellulose nanofibril-reinforced hydrogel with robust mechanical, self-healing, pH-responsive and antibacterial characteristics for wound dressing applications
Source: J Nanobiotechnology. 2022 Jul 6;20:312. doi: 10.1186/s12951-022-01523-5 (PMC9258071; doi:10.1186/s12951-022-01523-5)
Supplement: Supplementary file 1 — Additional file 1: Fig. S1. FTIR spectra of PEG, RSV, CNF and RSV-PEG-CNF conjugate. Fig. S2. Swelling ratio of different hydrogel groups. Fig. S3. Water vapor permeability of control, commercial Tegaderm film and RPC/PB hydrogel groups with different RPC content. Fig. S4. SEM images of RPC conjugate, PB, RPC/PB-0.2, RPC/PB-0.5 and RPC/PB-0.8 hydrogels. Fig. S5. Storage modulus (G') and loss modulus (G'') of PB, RPC/PB-0.2, RPC/PB-0.5 and RPC/PB-0.8 hydrogels versus frequency. Fig. S6. RSV release profiles from RPC conjugate under pH 5.4, 6.2 and 7.4. Fig. S7. FTIR spectra of PB, C/PB-0.5 and RPC/PB-0.5 hydrogels. [file 12951_2022_1523_MOESM1_ESM.docx]

**A cellulose nanofibril-reinforced hydrogel with robust mechanical, self-healing, pH-responsive and antibacterial characteristics for wound dressing applications**

Guihua Yang ^a, ‡^, Zhikun Zhang ^a, ‡^, Kefeng Liu ^a,*^, Xingxiang Ji ^a^, Pedram Fatehi ^a,b^, Jiachuan Chen ^a,*^

*^a^ State Key Laboratory of Biobased Material and Green Papermaking, Qilu University of Technology (Shandong Academy of Sciences), Jinan, Shandong, 250353, China*

*^b^ Biorefining Research Institute and Chemical Engineering Department, Lakehead University, Thunder Bay, ON, Canada*

** Corresponding authors. E-mail addresses: kfliu@qlu.edu.cn; chenjc@qlu.edu.cn*

‡ These authors contributed equally to this work.

**Methods**

**Preparation of TOCNFs**

TOCNFs were prepared using the previous methods in literature with slight modifications. Briefly, 10.0 g bleached softwood kraft pulp (BSKP) was suspended in 600 mL of DI water, followed by mechanical stirring for 30 min. TEMPO (0.16 g) and NaBr (1.0 g) were also dissolved in 400 mL DI water with magnetic stirring for 1 h in a beaker and then the solution was added slowly into the pulp solution. After that, a certain quantity of NaClO (10 mmol/g fiber) was added slowly into the above mixed solution for the oxidation reaction. The pH value of the mixture remained at 10.5 by adding 0.5 M NaOH. Ethanol was used to terminate the oxidation reaction. The product was washed thoroughly with DI water four times. Finally, the product with the concentration of 0.1 wt.% was homogenized by a GYB40-10S high-pressure homogenizer for 4 times at 400 bar and then concentrated by a rotary evaporator to the concentration of 0.2 wt.%, 0.5 wt.%, 0.8 wt.%, and 1.0 wt.%. The surface carboxyl content of prepared TOCNFs is measured by conductivity titration method (1.4 mmol/g).


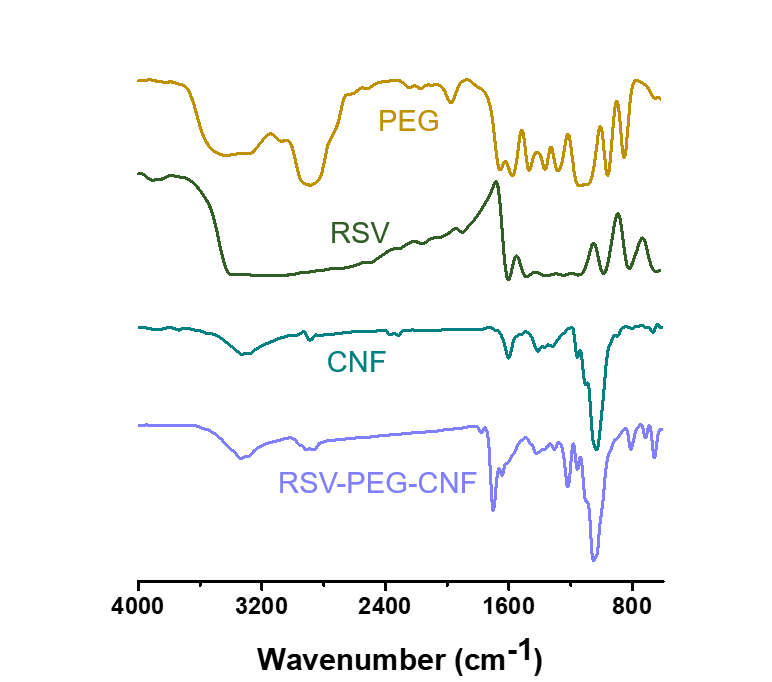


**Fig. S1.** FTIR spectra of PEG, RSV, CNF and RSV-PEG-CNF conjugate.


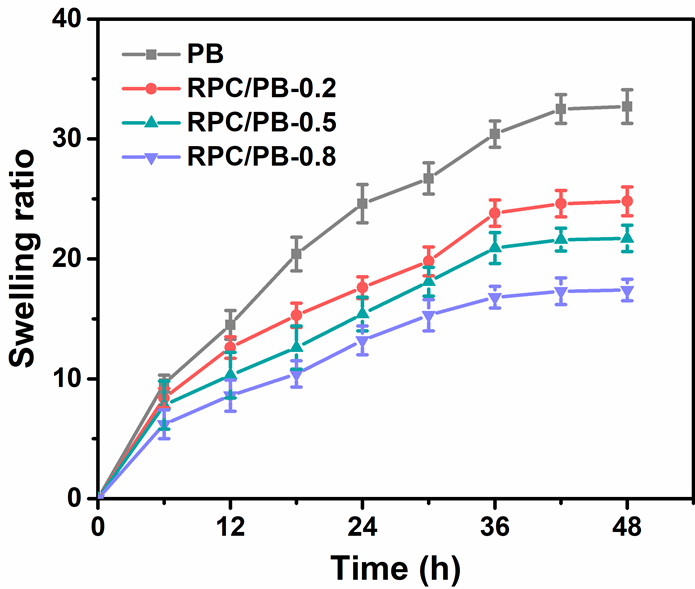


**Fig. S2.** Swelling ratio of different hydrogel groups.


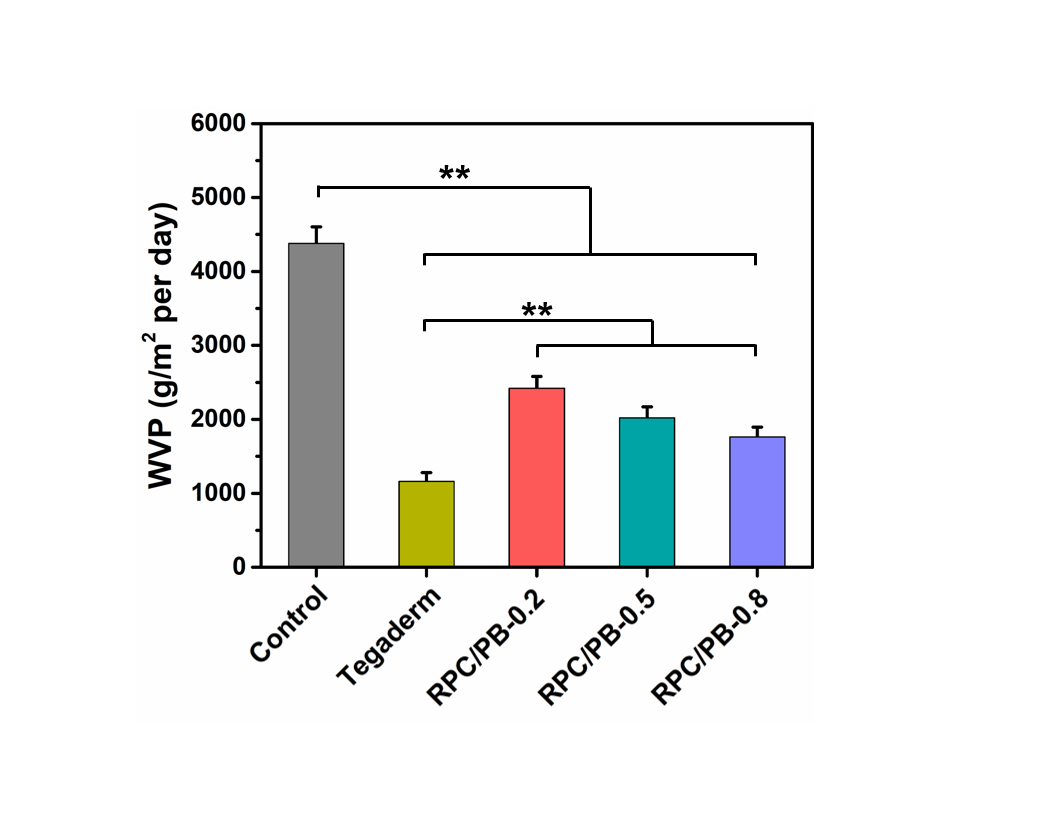


**Fig. S3.** Water vapor permeability of control (no covering), commercial Tegaderm film and RPC/PB hydrogel groups with different RPC content.


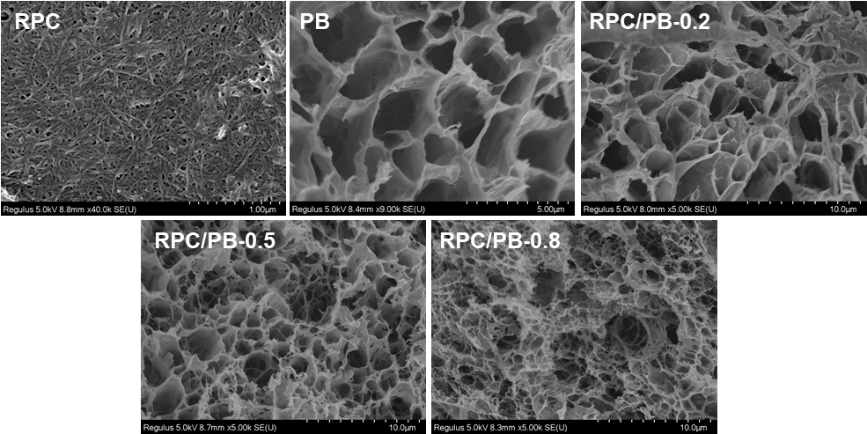


**Fig. S4** SEM images of RPC conjugate, PB, RPC/PB-0.2, RPC/PB-0.5 and RPC/PB-0.8 hydrogels.


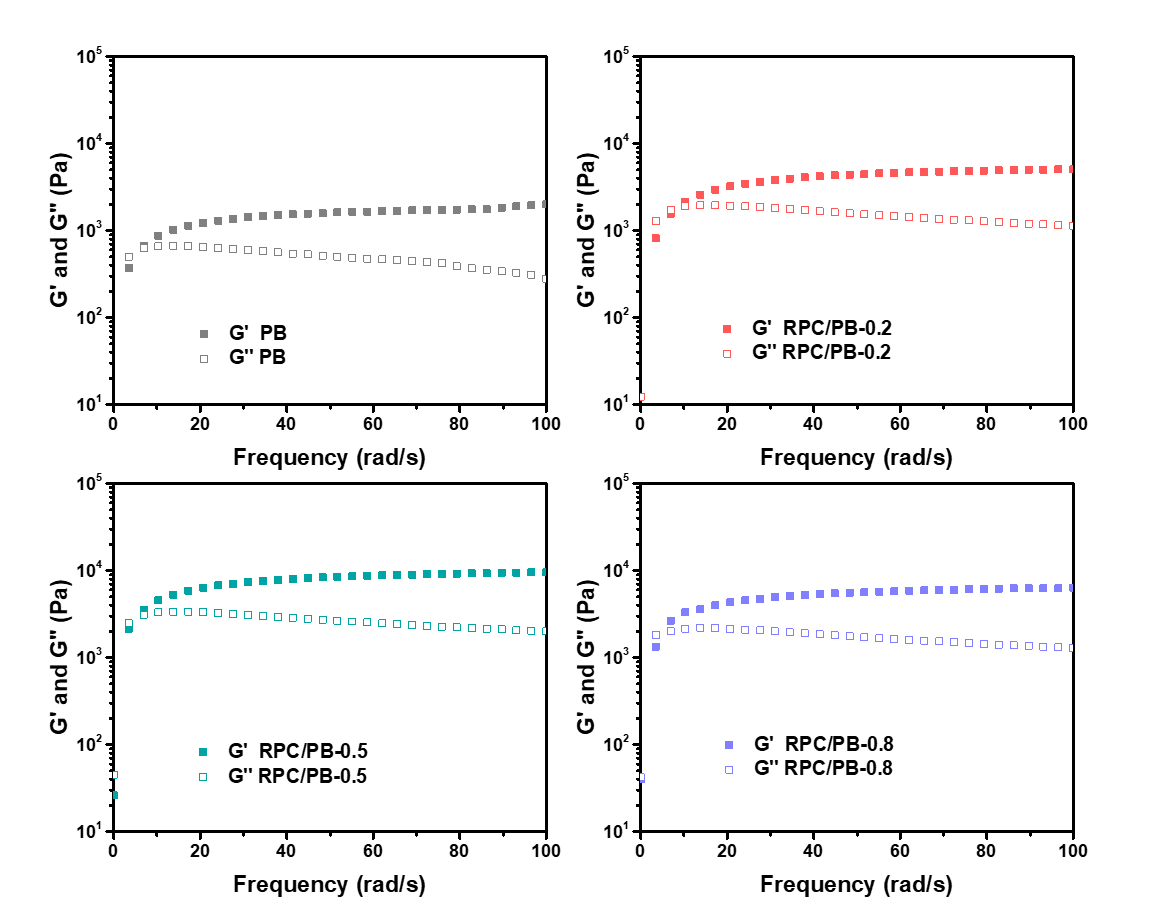


**Fig. S5** Storage modulus (G’) and loss modulus (G’’) of PB, RPC/PB-0.2, RPC/PB-0.5 and RPC/PB-0.8 hydrogels versus frequency.





**Fig. S6** In vitro RSV release profiles from RPC conjugate under pH 5.4, 6.2 and 7.4.


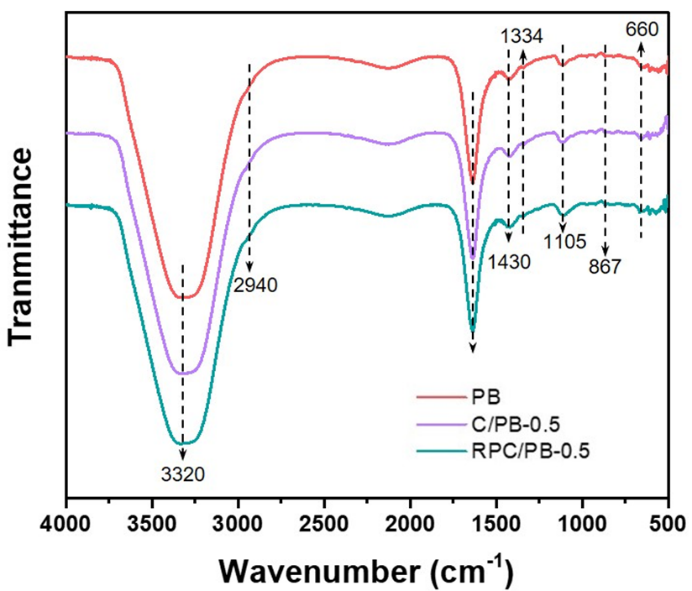


**Fig. S7** FTIR spectra of PB, C/PB-0.5 and RPC/PB-0.5 hydrogels.

As shown in Fig. S7, a large peak attributed to O-H stretching was observed at 3320 cm^-1^ in all spectra. This broad band corresponded to the -OH groups of PVA or nanocellulose that had complexed with borate ions during the assembly process [1]. The absorption peak at 1105 cm^-1^ was assigned to the C–O in stretching mode for PVA [2]. Moreover, the hydrogels displayed several characteristic peaks of borax and borate, including 1428 cm^-1^ and 1334 cm^-1^ (asymmetric stretching relaxation of B-O-C), 867 cm^-1^ (B-O stretching from residual B(OH)_4_^-^), demonstrating the multi-complexation and crosslinking between CNF, PVA chains, and borate [3].

1. Manna U, Patil S: **Borax mediated layer-by-layer self-assembly of neutral poly(vinyl alcohol) and chitosan.** *Journal of Physical Chemistry B* 2009, **113:**9137-9142.

2. Taleb M, El-Mohdy H, El-Rehim H: **Radiation preparation of PVA/CMC copolymers and their application in removal of dyes.** *Journal of hazardous materials* 2009, **168:**68-75.

3. Spoljaric S, Salminen A, Luong ND, Sepp L J: **Stable, self-healing hydrogels from nanofibrillated cellulose, poly(vinyl alcohol) and borax via reversible crosslinking.** *European Polymer Journal* 2014, **56:**105-117.
